# cBAF and MYC: decision makers for T cell memory differentiation

**DOI:** 10.1038/s41392-022-01238-x

**Published:** 2022-12-12

**Authors:** Kaili Ma, Hongcheng Cheng, Lianjun Zhang

**Affiliations:** 1 CAMS Key Laboratory of Synthetic Biology Regulatory Element, Institute of Systems Medicine, Chinese Academy of Medical Sciences and Peking Union Medical College, 100005 Beijing, China; 2 Suzhou Institute of Systems Medicine, Suzhou, 215123 Jiangsu China; 3 Jiangsu Center for the Collaboration and Innovation of Cancer Biotherapy, Cancer Institute, Xuzhou Medical University, Xuzhou, China

**Keywords:** Adaptive immunity, Lymphocytes

**Fig. 1:**
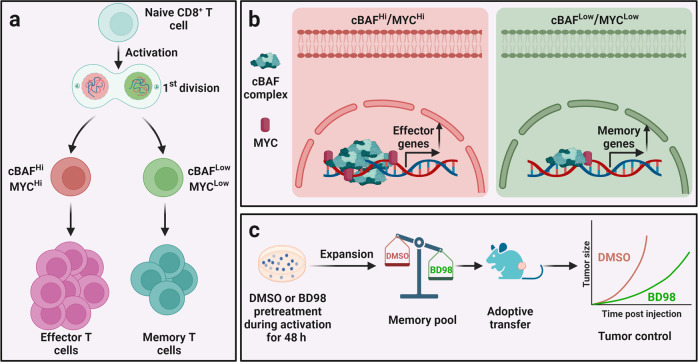
Asymmetrical division of cBAF complex and MYC dictates CD8^+^ T cell fate decision. **a** During the first division of naïve CD8^+^ T cells, daughter cells with low MYC and low cBAF preferentially differentiate towards T_mem_ fate. **b** MYC interacts with the cBAF complex and promotes T_eff_ and suppress T_mem_ fate trajectories. **c** Transient inhibition of cBAF activity during CD8^+^ T cell priming by BD98 (BRD-K98645985) promotes the expansion of memory pool and enhances their anti-tumor capacity. Figure was created with BioRender.com (https://biorender.com/)

In a recent paper published in *Nature*, Guo et al.^1^ demonstrated that asymmetrically segregated MYC and canonical BRG1/BRM-associated factor (cBAF) at first division of activated CD8^+^ T cells cooperate to dictate T cell fate by remodeling of epigenetics and chromatin state. Further pharmacological or genetic inhibition of cBAF in chimeric antigen receptor T (CAR-T) cells considerably promotes their anti-tumor capacity in vivo, presumably by biased memory differentiation post transfer (Fig. 1).

Accumulating evidence suggests that T cell effector or memory differentiation is highly coordinated via metabolic and epigenetic reprogramming.^2^ To characterize the key epigenetic regulators involved in T cell memory differentiation, Guo et al. carried out a CRISPR screen and identified several components of the cBAF complex as key negative regulators of T cell memory differentiation in response to acute infection,^1^ which is also confirmed by a parallel study showing that deficiency of ARID1A, the critical component of cBAF complex, enhances CD8^+^ T cell persistence and anti-tumor T cell function.^3^

The mammalian BAF (SWI/SNF family) chromatin remodeling complexes exist in three distinct assemblies: cBAF, polybromo-associated BAF (PBAF) and noncanonical BAF (ncBAF). They all contain one of two mutually exclusive ATPase subunits, either BRG1 or BRM. To probe the roles of different BAF complex subunits in T cell differentiation, several model systems harboring T cells with deficiency of different BAF subunit were generated. Deletion of BAF component SMARCD2 or the cBAF-specific component ARID1A in CD8^+^ T cells increase T_mem_ formation, as demonstrated by high expression of memory-associated surface markers CD62L and CD127, the enrichment of T_mem_ gene signatures and the increased proliferation potential and higher effector function upon antigen re-challenge. Ablation of the shared components SMARCC1 or ATPase subunit BRG1, which is present in cBAF, PBAF and ncBAF complexes, also promotes T_mem_ generation and suppresses T_eff_ differentiation. In contrast, deficiency of the PBAF-specific component PBRM1 or the ncBAF components BRD9 and BICRA does not impact memory precursor (MP) generation in vivo. Thus, these findings demonstrated the specific role of BRG1-containing cBAF complex in antagonizing the memory T cell fate decision.

Intriguingly, the CRISPR screen also identified MYC as a key regulator of MP differentiation beyond cBAF complex. A previous study has demonstrated that asymmetric distribution of the transcription factor c-MYC during the first division of activated T cells greatly impacted the transcriptional programs of daughter T cells, and c-MYC^high^ daughter cells are more prone to differentiate into T_eff_ cells, whereas c-MYC^low^ cells preferentially acquire T_mem_ fate in vivo.^4^ To better characterize the interplay between MYC and cBAF complex at early time points after T-cell-receptor (TCR) stimulation, the distribution pattern of the cBAF components SMARCB1, ARID1A and BRG1 in first-division CD8^+^ T cells was measured. Surprisingly, the authors found that cBAF components and MYC were frequently co-assorted asymmetrically to the two daughter cells. Using assay of transposase-accessible chromatin with visualization (ATAC-see) and assay for transposase-accessible chromatin with high-throughput sequencing (ATAC-seq), they further revealed that first-division MYC^low^ cells exhibited reduced chromatin accessibility and enhanced T_mem_ cell signatures. Ablation of the ARID1A promotes the differentiation of MP subset both in MYC^low^ and MYC^high^ cells, indicating that cBAF likely functions downstream of MYC to co-opt T-cell fate decisions. Notably, the cBAF components ARID1A and SMARCB1 co-precipitated with MYC. The CUT&RUN assay further reveals that MYC occupies 45% of ARID1A-binding sites and 42% of BRG1-binding sites, which supports the expression of MYC-target gene sets including those critically required for T-cell activation and differentiation, and effector-associated molecules. Furthermore, deficiency of ARID1A remarkably reduces the chromatin binding of MYC to gene elements that are important to T_eff_ differentiation. Similarly, reduced MYC levels in *Myc*^+/−^ CD8^+^ T cells also largely impairs ARID1A and BRG1 chromatin binding, suggesting that MYC and cBAF physically interact and act in coordination to foster T_eff_ cell fate decision.

The differentiation of CD8^+^ T cells into T_mem_ cells instead of T_eff_ cells is tightly related to the success of T cell-based immunotherapies. Cancer patients greatly benefit from the adoptive transfer of T cells with long-term memory potential.^5^ Not surprisingly, genetic deletion of SMARCD2 (a BAF component) or pharmacological inhibition of cBAF by BRD-K98645985 (BD98) in OT-1 cells showed greatly improved tumor control using either B16-OVA or MC38-OVA tumor models. Furthermore, transient pre-conditioning of mouse CAR-T cells against B7-H3 with BD98 during the initial activation stage markedly enhanced their anti-tumor efficacy with several mouse solid tumor models. Similar findings were reproduced with human CD8^+^ T cells upon BD98 transient treatment, implying that pharmacological manipulation of cBAF activity in T cells has great potential for improved adoptive T cell therapy.

Elucidating the cellular and molecular mechanisms underlying T cell memory formation remains the critical question in the field of T cell immunology, which would provide the rationale for the development of effective prophylactic and therapeutic approaches against infection and cancer. Several models have been proposed to explain the effector/memory T cell lineage commitment though considerable controversy remains. The present study not only provides compelling evidence to support a novel model of CD8^+^ T cell fate determination into effector or memory trajectories but also has important clinical implications. First, asymmetric cell division seems to dictate distinct T cell fate. Of note, around 5% of activated T cells adopt the memory differentiation, and it is likely that only a small fraction of the first round activated T cells undergoes asymmetric cell division. Though the intensity or duration of T cell activation or signaling may be linked to amino acid metabolism and mTORC1 activity,^4^ which is believed to activate MYC subsequently, the precise initial signal triggering the differential segregation of MYC as well as the components of cBAF complex remains unknown. Second, it awaits further investigations on how cBAF complex and MYC were asymmetrically distributed to two daughter cells arising from the same activated T cell during the first cell division and how cellular cargo proteins were coordinated to segregate those key components into two distinct daughter cells? Clearly, the cBAF complex plays critical roles in finally “locking” the committed T cell fate. Last but not least, the observations that either genetic or pharmacological inhibition of the cBAF activity leads to superior anti-tumor T cell immunity will attract considerable attention for designing next-generation CAR-T and TCR-T cellular therapies.
